# Depression and aging: insights from brain age prediction models

**DOI:** 10.1017/S0033291726104851

**Published:** 2026-06-23

**Authors:** Orla Mitchell, Michael Connaughton, John R. Kelly, Andrew Harkin, Darren W. Roddy, Monica Aas

**Affiliations:** 1 Department of Psychiatry, Royal College of Surgeons in Ireland, Dublin 2, Ireland; 2 Trinity College Institute of Neuroscience, Trinity College Dublin, Dublin 2, Ireland; 3 Social, Genetic & Developmental Psychiatry Centre, Institute of Psychiatry, Psychology & Neuroscience, King’s College London, London, United Kingdom

**Keywords:** brain age, brain-PAD, HPA axis, major depressive disorder, neuroimaging

## Abstract

**Background:**

The impact of depression on brain aging remains unclear, but both have been linked to stressful life events. Shared biological pathways may underlie structural brain changes. Clarifying these relationships could advance understanding of underlying mechanisms and inform treatment approaches.

**Methods:**

Structural MRI scans of 190 participants (controls, *n* = 110, clinically diagnosed with major depressive disorder [MDD], *n* = 80), from the REDEEM dataset, were input into three pretrained brain age prediction models: brainageR, DeepBrainNet, and pyment. Prediction accuracy was compared in controls to identify the optimal model. DeepBrainNet demonstrated the highest accuracy and was selected for subsequent analysis. Brain-predicted age difference (brain-PAD) was calculated as predicted age minus chronological age. Linear regression examined the effects of MDD diagnosis, childhood maltreatment, and cortisol awakening response on brain-PAD.

**Results:**

Depressed participants reported greater childhood maltreatment but a similar cortisol awakening response. An Age × Group interaction (*β* = 0.34, 95% CI: 0.15–0.53, *p* < 0.001) indicated older adults with MDD exhibited greater positive deviations from normative brain age predictions, suggesting nonuniform brain aging across the lifespan. Cortisol awakening response showed a negative association with brain-PAD (*β* = −0.01, 95% CI: −0.01 to −0.00, *p* = 0.041), indicating higher HPA-axis reactivity was linked to younger-appearing brains. Females showed lower brain-PAD than males, reflecting younger-appearing brains.

**Conclusions:**

MDD was associated with age-dependent differences in brain-PAD. The protective association between cortisol awakening response and brain age highlights the importance of integrating stress biomarkers to better understand neural aging mechanisms in depression.

## Introduction

Aging, a major risk factor for many adverse health outcomes, is marked by progressive, time-dependent functional decline, driven by the accumulation of cellular damage (López-Otín et al., 2013). This gradual and irreversible process affects much of the body’s functioning, including the brain’s, commonly presenting as declines in executive function and deficits in working and episodic memory (Alexander et al., 2012). However, brain aging is highly heterogeneous (Eavani et al., 2018). Neuroimaging-derived brain age prediction has emerged as an informative biomarker of brain health (Cole & Franke, 2017; Cole et al., 2017; Franke, Ziegler, Klöppel, & Gaser, 2010), with environmental, genetic, and lifestyle factors all influencing aging rates. Growing evidence suggests that psychopathology may play a role in this heterogeneity (Cole, Marioni, Harris, & Deary, 2019).

Brain age prediction models use machine learning algorithms to develop predictive frameworks that can estimate an individual’s brain age based on structural MRI features, such as cortical and subcortical volumes and thickness (Valizadeh, Hänggi, Mérillat, & Jäncke, 2017). These models learn patterns between the neuroanatomical data and chronological age labels to build regression frameworks (Cole et al., 2017), which can then be applied to new data to predict brain age. The resulting difference between predicted and chronological age is called brain-predicted age difference (brain-PAD), with higher scores typically indicating accelerated brain aging. Brain-PAD has demonstrated functional relevance through associations with clinical outcomes across various disorders, including schizophrenia, mild cognitive impairment, and dementia, with increased brain-PAD consistently linked to poorer clinical outcomes (Kaufmann et al., 2019).

Although accelerated brain aging has been consistently associated with psychotic disorders (Ballester et al., 2022), findings in major depressive disorder (MDD) remain inconsistent. These discrepancies suggest that the relationship between MDD and brain aging may be influenced by additional biological and environmental factors. While some effects may arise indirectly through depressive symptoms, such as disrupted sleep and reduced cognitive engagement (Carroll & Prather, 2021), emerging evidence suggests that shared biological mechanisms may also contribute to altered aging trajectories. Throughout this manuscript, the term ‘depression’ refers to individuals with a diagnosis of MDD.

The hypothalamic–pituitary–adrenal (HPA) axis is the body’s main stress response system, and HPA axis dysfunction is a key mechanism implicated in both depression and aging (de Souza et al., 2025) and can present as hypercortisolism, abnormal stress responsivity, or disrupted cortisol rhythms (Ring, 2025). Chronic stress and altered cortisol dynamics, common in MDD, are associated with structural brain changes and neurodegeneration (Ouanes & Popp, 2019). Early life adversity (ELA) might also contribute to structural brain alterations through lasting effects on stress–response systems, potentially compounding vulnerability to both depression and accelerated aging. While peripheral biomarkers of aging, such as telomere length and DNA methylation, have been linked to depression, neuroimaging-based estimates of brain age may provide a more direct and integrative measure of neural aging.

To the best of our knowledge, no study has examined the association among brain-PAD, cortisol, and ELA in depression. Moreover, inconsistent findings in the literature may reflect complex interactions with biological and social factors. The present study examined brain-PAD in clinically diagnosed depressed participants compared to controls. With MDD projected to become the leading contributor to global health burden by 2030 (Kessler & Bromet, 2013), identifying neuroimaging biomarkers of disease progression may be critical for informing future intervention. Based on this evidence, we hypothesized that depressed participants would show higher brain-PAD than controls, and that this relationship might interact with age, reflecting cumulative neurobiological effects of repeated stress exposure on typical aging processes, leading to increasingly divergent brain aging trajectories over time. We further hypothesized that the depressed group would show greater childhood trauma exposure and higher cortisol awakening response, and that both would be positively associated with brain-PAD.

## Methods and materials

### Participants

The study included 190 participants (controls, *n* = 110; depressed, *n* = 80) from the REDEEM (Research in Depression: Endocrinology, Epigenetics and neuroiMaging) study at Trinity College Dublin, a project investigating biological and neuroimaging mechanisms underlying MDD. Participants with depression were recruited through HSE Psychiatry Services, and controls were willing participants from the general population, recruited through local advertisements. Exclusion criteria for the depression group included psychotic or substance use disorders, chronic medical illness, steroid medication use, and MRI contraindications. Controls were required to have no history of psychiatric or chronic medical conditions and no steroid medication use. Group demographics are presented in Table 1. Ethical approval was granted by the Tallaght Hospital/St. James’s Hospital Joint Research Ethics Committee (2013/23/02).

**Table 1.** Demographic and clinical characteristics Table 1. long description.

		All subjects(*n* = 190)	Controls(*n* = 110)	Depressed(*n* = 80)	Comparison between groups
		Mean (SD)	Mean (SD)	Mean (SD)	*W* (*p*-value)
Age		32.4 (11.8)	31.5 (11.7)	33.5 (11.8)	3919 (0.2)
	BrainageR	32.7 (12.5)	32.6 (12.2)	32.7 (12.9)	4206 (0.96)
	DeepBrainNet	36.9 (10.8)	36.9 (10.5)	36.9 (11.4)	4499 (0.79)
	pyment	33.1 (8.4)	32.8 (8.9)	33.4 (7.7)	3976 (0.56)
Brain-PAD					
	BrainageR	0.44 (10.8)	1.1 (12.4)	−0.46 (8.2)	4591 (0.26)
	DeepBrainNet	4.61 (9.4)	5.5 (10.8)	3.5 (6.83)	5132 (0.051)
	pyment	0.85 (10.7)	1.3 (12)	0.24 (8.8)	4686 (0.17)
AUCi		(*n* = 53) 45 (357)	(*n* = 26) 88.6 (214)	(*n* = 30) 7.32 (445)	392 (0.6177)

		% (*n*)	% (*n*)	% (*n*)	*X* ^2^ (*p*-value)
Sex (males)		48.9 (93)	58.2 (64)	36.3 (29)	8.1 (0.0045) **
Ethnicity (White Irish)		84.7 (161)	82.7 (91)	87.5 (70)	0.36 (Fisher’s Exact)
Education status	Jr. Cert.	9.5 (18)	5.5 (6)	15 (12)	20.98 (1.06 × 10^−4^)***
	Leaving Cert.	20 (38)	13.6 (15)	28.8 (23)	
	Some college	23.2 (44)	20 (22)	27.5 (22)	
	College degree	47.4 (90)	60.9 (67)	28.8 (23)	
Employment status	Student	24.7 (47)	28.2 (31)	20 (16)	9.36 × 10^−8^ *** (Fisher’s Exact)
	Unemployed	7.9 (15)	0 (0)	18.8 (15)	
	On leave	16.8 (32)	10.9 (12)	25 (20)	
	Employed PT	0.5 (1)	0 (0)	1.2 (1)	
	Employed FT	47.9 (91)	58.2 (64)	33.8 (27)	
	Retired	2.1 (4)	2.7 (3)	1.2 (1)	
Marital status	Single	49.5 (95)	54.5 (60)	42.5 (34)	8.8 (0.032)*
	In partnership	18.4 (35)	21.8 (24)	13.8 (11)	
	Married	25.8 (49)	19.1 (21)	35 (28)	
	Separated	6.3 (12)	4.5 (5)	8.8 (7)	
Childhood Trauma Questionnaire		24.2 (46)	10 (11)	43.8 (35)	26.9 (2.1 × 10^−7^)***
Medication	None	66.3 (126)	–	20 (16)	–
	Antidepressant	18.4 (35)	–	43.8 (35)	
	Other psychotropic	1.1 (2)	–	2.5 (2)	
	Antidepressant + Other	14.2 (27)	–	33.8 (27)	

*Note:* For age, brain-PAD and AUCi group comparisons were conducted using the Wilcoxon rank-sum test. For categorical variables (sex, ethnicity, education, employment, marital status, and childhood trauma questionnaire), chi-squared tests were used; Fisher’s exact test was applied where expected cell sizes were small. Group-wise comparisons of medication use were not performed as the control group is unmedicated by design. AUCi, area under curve with respect to increase; Employed FT, employed full-time; Employed PT, employed part-time; Jr. Cert., junior certificate; Leaving Cert., leaving certificate; PAD, predicted age difference; SD, standard deviation. * *p* < 0.05, ** *p* < 0.01, *** *p* < 0.001.

### Clinical assessment

#### Depression

All patients with depression were screened for eligibility by a consultant psychiatrist based on criteria for a major depressive episode (MDE) from the Mini International Neuropsychiatric Interview (M.I.N.I) (Sheehan et al., 1998) and scored > 17 on the Hamilton Depression Rating 21-Item Scale (HAM-D-21) (Hamilton, 1960), a clinician-administered measure of depressive symptom severity, indicative of moderate depression. Control participants had to score ≤7 on the HAM-D-21. Episode type was recorded as part of the clinical interview. Participants were classified as first-episode if they were presenting with their first major depressive episode, or recurrent if they reported two or more previous depressive episodes. This distinction was used in exploratory analyses to examine whether relationships with brain-PAD differed depending on chronicity.

#### Childhood maltreatment

Childhood maltreatment exposure was assessed using the Childhood Trauma Questionnaire (CTQ) (Bernstein et al., 2003), a 25-item instrument that evaluates five subtypes of childhood maltreatment: physical, emotional, and sexual abuse, and physical and emotional neglect. We applied the moderate-to-severe cutoff thresholds published by the original authors to create a binary exposure variable (Bernstein et al., 2003). Participants who met the cutoff criteria for any of the five maltreatment categories received a score of 1, indicating exposure to childhood maltreatment. The CTQ includes a three-item Minimization/Denial Scale designed to detect underreporting of maltreatment experiences (reliability = 0.77) (MacDonald et al., 2016; MacDonald, Thomas, MacDonald, & Sciolla, 2015). This scale uses reverse-scored items that assess tendencies to minimize adverse experiences, such as reporting a ‘perfect childhood’ or stating they would not change anything about their family. Participants scoring ‘highly likely’ on any minimization/denial item received a score of 1, with a maximum possible score of 3. The CTQ is a retrospective self-report measure, and scores reflect perceived or recalled childhood adversity rather than independently verified environmental exposure. However, retrospective reports may capture subjective appraisal that is particularly relevant to psychopathology (Baldwin, Coleman, Francis, & Danese, 2024).

### MRI acquisition

#### Acquisition and quality control

All MRI scans were acquired using a Philips Intera Achieva 3 T MR system (32-channel head coil) at Trinity College Institute of Neuroscience, Dublin. High-resolution T1-weighted anatomical images were obtained (180 axial slices, T1W-IR1150 sequence, TE = 3.8 ms, TR = 8.4 ms, FOV 230 mm, 0.898 × 0.898 mm^2^, in-plane resolution, slice thickness 0.9 mm, flip angle alpha = 8°). Each of the three brain age prediction models required unique preprocessing, outlined in the Supplementary Material. All raw and preprocessed images were visually inspected for pathology and image quality.

### Brain age prediction

Three pretrained machine learning pipelines were applied: brainageR (v2.1), DeepBrainNet, and pyment. These models were selected based on evidence in the literature, with brainageR and pyment demonstrating the highest accuracy and test–retest reliability (Dörfel et al., 2023), while DeepBrainNet provided the recommended higher-complexity CNN approach for comparison (Joo et al., 2023; Seitz-Holland et al., 2024). All models were trained on large, multisite samples of healthy individuals, establishing normative brain aging trajectories, and are widely adopted in clinical neuroimaging (Clausen et al., 2022; Kim et al., 2024; Valdes-Hernandez et al., 2023). Deviations from normative predictions in clinical populations are quantified as brain-PAD. BrainageR employs Gaussian Process Regression on gray matter, white matter, and CSF probability maps (Cole et al., 2018), and was trained on 3,377 healthy individuals (mean age = 40.6 years, SD = 21.4, range = 18–92 years), from seven publicly available datasets. DeepBrainNet uses a 2D Convolution Neural Network (CNN) architecture applied to FreeSurfer-derived features across 80 axial slices (Bashyam et al., 2020) and was trained on 11,729 individuals aged 3–95 years (Valdes-Hernandez et al., 2023), with model performance evaluated on an independent test set of 2,739 individuals. Pyment applies a Simple Fully Convolution Network to 3D T1-weighted images (Peng et al., 2021). We selected the SFCN-reg model trained on 53,542 participants aged 3–95 years from 21 nonoverlapping publicly available datasets, which employs the base SFCN with a regression prediction head. Full technical details and preprocessing pipelines are provided in the Supplementary Material.

### Salivary cortisol analysis

Saliva samples were collected within 1 week of recruitment. Participants were instructed to avoid eating, drinking, or brushing teeth for 30 minutes before collection. Samples were collected in Salivette® tubes at three time points: 0, 30, and 60 minutes after waking, to assess cortisol awakening response (CAR). Cortisol concentrations were measured using liquid chromatography–tandem mass spectrometry (LC–MS/MS) as previously described (Jones, Owen, Adaway, & Keevil, 2012). CAR was calculated as the area under the curve with respect to increase (AUC*i*) using the equations below. This approach simplifies statistical analysis while preserving information from repeated measures (Pruessner, Hellhammer, Pruessner, & Lupien, 2003).

(1) $$\mathrm{AUC}g=\sum_{i=1}^{n-1}\frac{\left(m_{i+1}+m_{i}\right)}{2}$$

(2) $$\mathrm{AUC}i=AUCg-\left(n-1\right)*m_{1}$$

### Statistical analysis

Model performance was evaluated using several complementary metrics commonly reported in brain age prediction studies (Clausen et al., 2022). Pearson’s correlation coefficient (*r*) and the coefficient of determination (*R* ^2^) quantify the strength of association and proportion of variance in chronological age explained by predicted brain age. The intraclass correlation coefficient (ICC) provides a measure of agreement between predicted and chronological age, reflecting model reliability beyond simple correlation. Prediction error was assessed using mean absolute error (MAE) and root mean square error (RMSE), which quantify the average magnitude of prediction error, with RMSE placing greater weight on larger deviations. Together, these metrics provide a comprehensive evaluation of model accuracy, agreement, and prediction error. The optimal model was that which demonstrated the highest ICC, *R* ^2^, and Pearson’s correlation values and the lowest MAE and RMSE. No cross-validation or model fitting was performed within the study sample. The full model selection procedure is outlined in the Supplementary Material.

Group demographics are presented in Table 1. Between-group differences in continuous variables (age, brain-PAD, and AUCi) were tested using Wilcoxon rank-sum tests, and categorical variables (sex, ethnicity, education status, employment status, marital status, and childhood trauma exposure) were tested using chi-squared tests, with Fisher’s exact test applied when expected cell counts were small. Medication comparisons were not conducted as the control group was unmedicated by design.

The primary analysis examined brain-PAD (predicted age–chronological age) differences between groups, using linear regression. Diagnostic plots indicated that model assumptions were adequately met. Age, which was mean-centered, and sex, both known influencers of brain age, were included as covariates (Le et al., 2018; Liang, Zhang, & Niu, 2019). We also examined episode type (first vs. recurrent) and tested Age × Group interactions to assess whether the association between age and brain-PAD differed between groups. Ethnicity did not improve model fit. False discovery rate (FDR) correction was applied to all models. Full model specifications are provided in the Supplementary Material.

We investigated associations between brain-PAD and stress-related variables (childhood maltreatment and CAR). Cortisol analyses were limited to participants with complete data (*n* = 53). Associations between CTQ measures, baseline cortisol, and AUCi were assessed using Spearman correlations. Sensitivity analyses were also conducted using continuous CTQ total and subtype scores, abuse and neglect related composites, quadratic age terms, CTQ minimization/denial scores (Church et al., 2017), and education, marital, and employment status. These additional analyses did not materially alter results and were not retained in the primary models, and details can be found in the Supplementary Material. False discovery rate (FDR) correction was applied to all models.

To evaluate potential age-related bias in brain age predictions, we examined associations between chronological age and brain-PAD within each group. Brain age prediction models can exhibit regression to the mean effects, and therefore, we additionally conducted sensitivity analyses using an age-bias correction procedure as described in Beheshti, Nugent, Potvin, and Duchesne (2019). This method generates age-bias-corrected brain-PAD estimates, which were used to repeat the regression analysis.

## Results

### Samples

Data from 190 participants (controls, *n* = 110; depressed, *n* = 80) from the REDEEM study were included in the analysis. Participant demographics are summarized in Table 1. Compared to the depressed group, the control group had more males, higher levels of education, were more frequently employed, and differed in marital status. More individuals with depression reported exposure to childhood trauma than controls. Participants ranged in age from 18 to 67 years (mean = 32.7 years, SD = 11.5), representing a relatively young adult sample compared to the full lifespan ranges used to train the brain age models.

### Model selection

DeepBrainNet was selected as the optimal model. Model performance metrics are visualized in Table 2. Further information regarding the model selection process and DeepBrainNet model validation can be found in the Supplementary Material.

**Table 2.** Model performance assessment metrics Table 2. long description.

Model	ICC	*R* ^2^	Pearson’s *R*	MAE	RMSE
brainageR	0.453	0.206	0.454	8.84	12.4
DeepBrainNet	0.471	0.28	0.529	9.37	12.1
pyment	0.317	0.107	0.328	9.37	12.0

*Note:* ICC, intraclass correlation; MAE, mean absolute error; RMSE, root mean square error.

### Primary analysis

**Figure 1.**
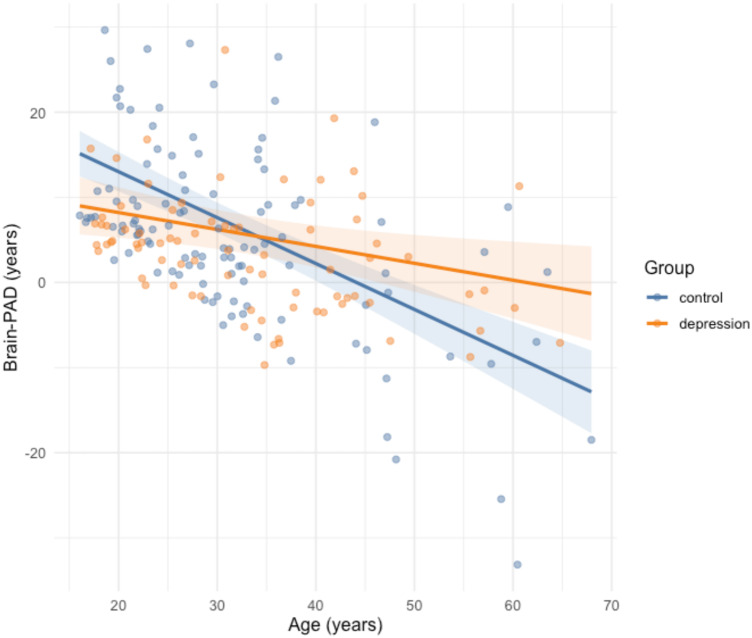
Interaction between brain-PAD and age by group. *Note:* Association between chronological age and brain-predicted age difference (brain-PAD) by diagnostic group. Scatterplots with fitted regression lines illustrate the relationship between age and brain-PAD in control participants (*n* = 110) and individuals with major depressive disorder (MDD; *n* = 80). A significant Age × Group interaction indicates that the association between age and brain-PAD differs between groups, with a less negative slope in the depression group. Figure 1. long description.

A significant Age × Group interaction emerged (Age × Group model: *β* = 0.34, 95% CI: 0.15–0.53, *p* < 0.001), indicating that the age-brain-PAD slope was less negative in the depression group (Figure 1). This interaction substantially improved model fit over the main-effects models. Sex was associated with brain-PAD, with females showing lower values, with this effect remaining robust when adjusting for age, group, childhood trauma exposure, and cortisol awakening response (Group main effect: *β* = −2.97, 95% CI: −5.34 to −0.61, *p* = 0.014; Age × Group Interaction: *β* = −3.26, 95% CI: −5.57 to −0.96, *p* = 0.006). However, no main effect of group emerged across models (Group main effect: *β* = −0.56, 95% CI: −2.96 to 1.84, *p* = 0.646; Age × Group interaction: *β* = −0.6, 95% CI: −2.93 to 1.73, *p* = 0.613). Within the depression group, clinical chronicity (recurrent vs. first episode) showed no main (Group subtype main effect: *β* = −0.58, 95% CI: −4.11 to 2.95, *p* = 0.745) or interaction effect (Age × Group subtype interaction: *β* = −0.13, 95% CI: −0.42 to 0.17, *p* = 0.397).

To evaluate potential age bias in the brain-PAD estimates, we examined associations between chronological age and brain-PAD within each group. Chronological age was negatively associated with brain-PAD in controls (*r* = −0.57, *p* < .001) and in depression (*r* = −0.36, *p* = .001), consistent with the expected regression to the mean phenomenon. Linear regression analyses similarly showed that age significantly predicted brain-PAD in controls (*β* = −0.53, *R* ^2^ = 0.32, *p* < .001) and more weakly in depression (*β* = −0.21, *R* ^2^ = 0.13, *p* = .001).

To determine whether this age dependence influenced the primary findings, we repeated the main analysis using age bias-corrected brain-PAD values. After correction, the association between age and brain-PAD was no longer present (*β* = −0.01, *p* = .84), indicating that the correction had removed the age bias. The Age × Group interaction, however, remained unchanged in magnitude and significance (*β* = 0.34, *p* = .0007), suggesting that the interaction effect is unlikely to be solely explained by regression to the mean bias in the brain age predictions (Supplementary Material).

### Secondary analysis

**Figure 2.**
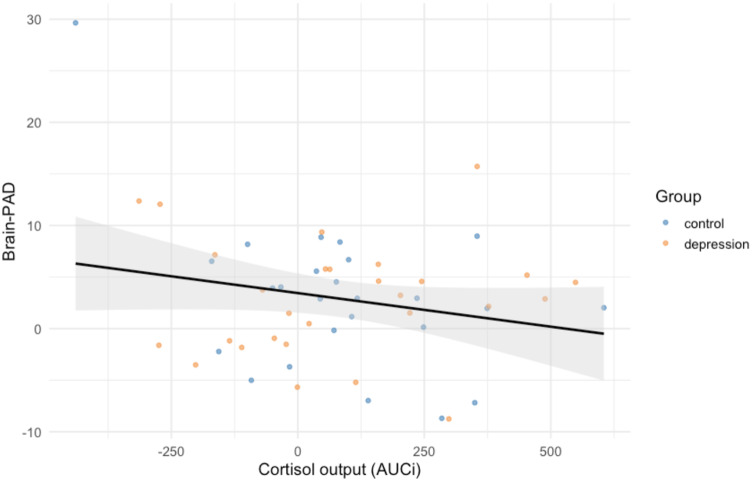
Association between cortisol output and brain-PAD. *Note:* Association between cortisol awakening response (CAR) and brain-predicted age difference (brain-PAD). Scatterplot showing the relationship between CAR (area under the curve with respect to increase; AUCi) and brain-PAD (*n* = 53). The fitted regression line represents the overall association across participants, indicating that higher cortisol reactivity is associated with lower brain-PAD values. Figure 2. long description.

Our exploratory analyses examined whether distal environmental exposures and proximal biological stress markers were independently associated with brain-PAD. Childhood maltreatment, assessed retrospectively via the CTQ, represents a distal environmental exposure, while cortisol awakening response (CAR) reflects HPA-axis functioning and represents a biological index of stress physiology. These variables were therefore treated as conceptually distinct pathways through which early adversity and its downstream biological sequelae may relate to brain aging. CAR demonstrated a significant negative association with brain-PAD (CAR main effect: *β* = −0.01, 95% CI: −0.01 to 0.00, *p* = 0.041; Figure 2), indicating that higher cortisol reactivity was associated with lower brain-PAD (younger-appearing brains). This relationship appeared consistent across groups, as the AUCi × Group interaction was nonsignificant (CAR ×Group interaction: *β* = 0.01, 95% CI: −0.00 to 0.02, *p* = 0.134).

In contrast, childhood trauma exposure showed no significant association with brain-PAD (Childhood trauma main effect: *β* = 1.19, 95% CI: −1.50 to 3.87, *p* = 0.384). In the interaction model, the association between ELA exposure and brain-PAD in controls was nonsignificant (*β* = 4.69, 95% CI: −0.38 to 9.76, *p* = 0.07), and the CTQ × Group interaction was also nonsignificant (*β* = −4.46, 95% CI: −10.65 to 1.74, *p* = 0.16), indicating no reliable group differences in this association. We found no correlation between CTQ and baseline cortisol (*p* = 0.14) or CAR (*p* = 0.54). Full results of all models can be found in Table 3. Information regarding sensitivity analyses for different CTQ scoring can be found in the Supplementary Material.

**Table 3.** Multiple linear regression model results Table 3. long description.

	Beta	CI	t-value	P
Group main effect: AIC = 1337.7, BIC = 1354, N = 190, R^2^ = 0.273				
Intercept	6.36	4.56 to 8.17	6.94	<.001*
Age	−0.39	−0.49 to −0.29	−7.87	<.001*
Sex	−2.97	−5.34 to −0.61	−2.48	.014*
Group	−0.56	−2.96 to 1.84	−0.46	.646
Group subtype main effect: AIC = 531.1, BIC = 543, N = 80, R^2^ = 0.142				
Intercept	4.9	2.29 to 7.52	3.74	<.001*
Age	−0.19	−0.34 to −0.04	−2.58	.012*
Sex	−1.55	−4.58 to 1.47	−1.02	.31
Depression subgroup	−0.58	−4.11 to 2.95	−0.33	.745
Age × Group interaction effect: AIC = 1327.8, BIC = 1327.8, N = 190, R^2^ = 0.317				
Intercept	6.36	4.61 to 8.12	7.14	<.001*
Age	−0.54	−0.67 to −0.41	−8.38	<.001*
Group	−0.6	−2.93 to 1.73	−0.51	.613
Sex	−3.26	−5.57 to −0.96	−2.8	.006*
Age*Group	0.34	0.15 to 0.53	3.46	<.001*
Age × Group subtype interaction effect: AIC = 532.4, BIC = 546.6, N = 80, R^2^ = 0.150				
Intercept	5.31	2.52 to 8.09	3.8	<.001*
Age	−0.12	−0.34 to 0.09	−1.16	.248
Depression subtype	−0.28	−3.89 to 3.33	−0.15	.878
Sex	−1.77	−4.84 to 1.31	−1.15	.255
Age*Depression subtype	−0.13	−0.42 to 0.17	−0.85	.397
Childhood trauma main effect: AIC = 1337.2, BIC = 1353.4, N = 190, R^2^ = 0.275				
Intercept	5.91	4.15 to 7.67	6.61	<.001*
Age	−0.39	−0.49 to −0.30	−7.94	<.001*
Sex	−3.11	−5.41 to −0.80	−2.66	.008*
CTQ	1.19	−1.50 to 3.87	0.87	.384
CAR main effect: AIC = 336.8, BIC = 346.7, N = 53, R^2^ = 0.338				
Intercept	4.87	2.11 to 7.64	3.54	<.001*
Age	−0.31	−0.45 to −0.17	−4.57	<.001*
Sex	−1.88	−5.22 to 1.45	−1.14	.262
AUCi	−0.01	−0.01 to −0.00	−2.09	.041*
Childhood trauma × Group interaction effect: AIC = 1338.3, BIC = 1361, N = 190, R^2^ = 0.286				
Intercept	5.78	3.88 to 7.69	5.98	<.001*
Age	−0.39	−0.49 to −0.29	−7.81	<.001*
Sex	−2.7	−5.07 to −0.33	−2.24	.026*
CTQ	4.69	−0.38 to 9.76	1.82	.07
Group	−0.26	−3.16 to 2.63	−0.18	.859
CTQ*Group	−4.46	−10.65 to 1.74	−1.42	.157
CAR × Group interaction effect: AIC = 338.2, BIC = 352.0, N = 53, R^2^ = 0.369				
Intercept	5.34	1.77 to 8.92	3.01	.004*
Age	−0.29	−0.43 to −0.15	−4.13	<.001*
Sex	−1.68	−5.13 to 1.77	−0.98	.333
AUCi	−0.01	−0.02 to −0.00	−2.54	.014*
Group	−1.04	−4.39 to 2.30	−0.63	.534
AUCi*Group	0.01	−0.00 to 0.02	1.52	.134

*Note:* All reported p-values reflect tests corrected for multiple comparisons using the false discovery rate (FDR). AIC, Aikake Information Criterion; AUCi, area under curve with relation to increase (cortisol); BIC, Bayesian Information Criterion; CAR, cortisol awakening response; CTQ, Childhood Trauma Questionnaire; Depression subgroup, first episode versus recurrent depression; Group, clinical group (depression vs. control). * *p* < 0.05, ** *p* < 0.01, *** *p* < 0.001.

## Discussion

This study examined brain-predicted age difference (brain-PAD), the discrepancy between chronological age and predicted brain age, in clinically diagnosed depression. Our primary finding was a significant Age × Group interaction (*β* = 0.34, 95% CI: 0.15–0.53, *p* < 0.001), indicating that the age-related reduction in brain-PAD was attenuated in individuals with depression, compared to controls. This suggests that as individuals with depression age, they increasingly deviate from normative predictions (Figure 1), suggesting that the structural impact of depression may become more pronounced with advancing age.

Cortisol awakening response (CAR) showed a significant negative association with brain-PAD (*β* = −0.01, 95% CI: −0.01 to 0.00, *p* = 0.041), indicating that higher HPA-axis reactivity upon waking was associated with younger-appearing brains across both groups; however, this was an exploratory analysis, meaning results must be interpreted with caution. Childhood maltreatment (CTQ) showed only a trend-level association (*p* = 0.07), but individuals with depression reported significantly greater childhood trauma exposure than controls, consistent with established links between early life adversity and depression risk (McLaughlin et al., 2012). Across the models, a robust sex effect was observed. Female participants showed lower brain-PAD values than males, reflecting younger-appearing brains relative to chronological age. This difference remained after controlling for age, group, CTQ, and CAR, aligning with prior evidence that females exhibit younger-appearing brains based on both brain age prediction (Sanford et al., 2022) and metabolic brain age measures (Goyal et al., 2019). These findings suggest that sex-related biological factors may contribute to observed differences in brain-PAD between males and females.

Notably, we did not find a significant main effect of group (control vs. MDD) on brain-PAD, contrary to some previous reports (Besteher, Gaser, & Nenadić, 2019) and we established no significant differences between depression subgroups (first-episode vs. recurrent depression). However, our findings reveal a more nuanced relationship in which depression may modify the relationship between age and brain structure, highlighting the importance of considering how psychopathology interacts with normative aging. This age-dependent relationship challenges simplified models that assume consistent depression-related brain changes across the lifespan.

### Age-dependent brain aging in depression: Implications and mechanisms

The attenuation of typical age-related brain-PAD patterns in depression is a key finding of this study. Age emerged as the most consistent predictor of brain-PAD across seven of the eight models, with older participants showing more accurate predictions of brain age. However, the depression group exhibited a flatter age-brain-PAD slope. This pattern likely reflects the well-documented ‘regression to the mean’ phenomenon (Le et al., 2018; Liang et al., 2019), in which brain age prediction models systematically overestimate the ages of younger participants and underestimate the ages of older participants (Bland & Altman, 1994), which is widely recognized as an inherent feature, rather than a failure, of brain age prediction models (Butler et al., 2021). Although correction methods exist (Beheshti et al., 2019; Le et al., 2018; Smith et al., 2019), they require careful consideration and may not yield additional meaningful information (Butler et al., 2021). To evaluate whether this bias influenced our findings, we conducted a sensitivity analysis applying the age-bias correction procedure described by Beheshti et al. (2019), which removes systematic age-related residuals. As expected, this eliminated the association between chronological age and brain-PAD in controls, and importantly, when the primary regression models were repeated using these corrected values, the Age × Group interaction remained unchanged (*β* = 0.34, *p* = .0007). This suggests that the observed group difference in the age-brain-PAD association is unlikely to be solely explained by the regression to the mean phenomenon.

These age-related differences in the association between age and brain-PAD have important implications. They suggest that the relationship between depression and brain-PAD may vary across adulthood, with older individuals with depression showing greater positive deviations from normative brain age predictions. That is, a greater tendency for the brain to appear older than the chronological age, particularly in later adulthood. This pattern may be consistent with the possibility that depression-related structural brain differences become more apparent later in life, although longitudinal studies are required to determine whether such effects emerge or accumulate over time. These findings may help explain heterogeneity in previous brain age studies of depression and raise the possibility that older adults with depression represent a particularly vulnerable group. More broadly, the age-related differences observed here highlight the need for caution when interpreting cross-sectional findings and emphasize the value of longitudinal approaches.

### HPA-axis dysfunction and cortisol dynamics

The observed Age × Group interaction can be interpreted within the framework of allostatic load, which proposes that chronic stress can exert cumulative physiological effects on the brain (Guidi, Lucente, Sonino, & Fava, 2020). Within this framework, depression represents a chronic stressor that may relate to alterations in brain structure. Although the cross-sectional design prevents conclusions about temporal processes, the flatter age-brain-PAD slope observed in the depression group is broadly consistent with previous work linking greater exposure to psychopathology with larger brain-PAD estimates (Blake et al., 2023).

Dysregulation of the HPA-axis, characterized by elevated cortisol (Cosgriff, Abbott, Oakley-Browne, & Joyce, 1990; Nandam, Brazel, Zhou, & Jhaveri, 2019), reduced feedback sensitivity (Holsboer, 2000; Stamou, Colling, & Dichtel, 2023; Young et al., 2003), and flattened diurnal rhythms, is implicated in both depression and aging (Boehringer et al., 2015; Hsiao et al., 2010; Jiang et al., 2025; Nguyen et al., 2020). In this study, higher CAR was associated with younger-appearing brains across both groups, offering novel insight into how stress reactivity relates to brain aging. Although CAR alterations in depression are inconsistently reported (Hsiao et al., 2010; Pruessner et al., 2003), blunting, due to elevated baseline cortisol, may reflect chronic dysregulation (Adam et al., 2017). In our data, baseline cortisol was numerically higher in the depression group (11.0 vs. 7.5 nmol/L) but not significantly so (*p* = 0.094, 95% CI: −7.83 to 0.65), likely due to the modest sample size and high variability. Taken together, these findings suggest that variation in stress responsivity may relate to differences in brain-PAD, although these results should be interpreted cautiously and require replication in larger samples.

Beyond stress axis dysfunction, cortisol also exerts direct neurobiological effects, with chronic elevation linked to neurotoxicity (Lupien, Juster, Raymond, & Marin, 2018), white matter microstructure disruption (van der Meulen, Amaya, Dekkers, & Meijer, 2022), and suppression of brain-derived neurotrophic factor (BDNF) transcription (Chen, Lombès, & Le Menuet, 2017; Puhlmann et al., 2021; Suri & Vaidya, 2013). Early life adversity (ELA) is a well-established depression risk factor (McLaughlin et al., 2012) and has been linked to structural brain alterations (Mitchell, Roddy, & Connaughton, 2025; Pollok et al., 2022). In our sample, depressed individuals reported greater trauma exposure than controls (*p* < 0.001), and although CTQ binary exposure only showed a trend toward association with brain-PAD (*p* = 0.07), this pattern aligns with previous findings linking greater adversity to advanced brain-PADs (Hatton et al., 2018). Exposure to childhood trauma was also not associated with baseline cortisol or cortisol awakening response in this sample, indicating that trauma-related differences in HPA-axis reactivity were not detectable here, although the limited cortisol subsample may have reduced sensitivity to subtle effects.

### Strengths

A key strength of this study is the use of clinician-diagnosed depression, rather than symptom-based classification, which enhances clinical relevance and increases confidence that findings reflect genuine depressive pathology. Consequently, the sample is phenotypically robust, and the identified neural signatures are likely more reliable than those derived from symptom-based groupings. Integrating stress-related measures (cortisol dynamics and childhood maltreatment) with diagnostic and neuroimaging data provides a more comprehensive perspective on factors associated with depression. This study is, to the best of our knowledge, the first to demonstrate that individuals with depression show a distinct association between age and brain-PAD compared to healthy controls, specifically with more positive deviations from normative brain age predictions with advancing age.

### Limitations

Several limitations warrant consideration. First, as highlighted in the original article by Bashyam et al. (2020), DeepBrainNet’s MDD classifier did not converge during model development, potentially reflecting reduced sensitivity of brain-PAD estimates in depression and perhaps explaining the modest group effects. Brain-PAD reflects both physiological variation and prediction error, with noise disproportionately affecting smaller datasets. Beyond the regression-to-the-mean effects discussed above, differences between the present sample and the populations used to train the brain age models may also influence prediction accuracy and contribute to age-related patterns in brain-PAD. Medication exposure and factors such as psychological resilience may also influence brain structure and stress physiology, but these variables were not examined in detail in the present study. The modest sample size necessitates cautious interpretation, particularly for the cortisol and depression subgroup analyses, where sample sizes were further reduced and the cross-sectional design limits causal inference. Childhood trauma was assessed using the retrospective Childhood Trauma Questionnaire (CTQ), which is susceptible to recall bias. However, retrospective measures also capture subjective appraisals of experience, which may be more closely linked to psychopathology than prospectively measured adversity (Baldwin et al., 2024). Thus, while imperfect, the CTQ provides clinically meaningful information about perceived early adversity.

### Future directions

Future research should adopt longitudinal designs to establish whether accelerated brain aging precedes, coincides with, or follows depression onset, and whether treatment response relates to changes in brain-PAD. Incorporating additional biological markers alongside HPA-axis measures and neuroimaging could clarify mechanisms linking depression to brain aging, and a systematic assessment of medication history could help disentangle antidepressant effects on brain-PAD. Sex-specific mechanisms also warrant further investigation, particularly to determine whether depression influences brain-aging trajectories similarly in males and females.

## Conclusion

This study demonstrates that brain-PAD differs according to clinical status, with older individuals with depression exhibiting greater positive deviations from normative brain age predictions. The finding that greater cortisol awakening response (CAR) is associated with younger-appearing brains across groups suggests a relationship between stress responsivity and brain-PAD. Conversely, lower CAR may reflect maladaptive chronic HPA-axis dysregulation; however, all cortisol-related findings require confirmation in larger cohorts. These findings highlight potential links between stress physiology and brain aging markers in depression, though longitudinal investigations are required to clarify their temporal relationships.

## Supporting information

10.1017/S0033291726104851.sm001 Mitchell et al. supplementary material Mitchell et al. supplementary material

## Supplementary material

The supplementary material for this article can be found at http://doi.org/10.1017/S0033291726104851.

## Long descriptions

### Table 1. Long description

The table is organized into six columns: Characteristic, Sub-category, All subjects (n equals 190), Controls (n equals 110), Depressed (n equals 80), and Comparison between groups.

Key data points include:

* Age: Mean age is 31.5 for controls and 33.5 for depressed subjects (p equals 0.2).

* Brain-P A D (Predicted Age Difference): Measured via three models (BrainageR, DeepBrainNet, and pyment). DeepBrainNet shows the highest mean P A D in controls (5.5) versus depressed subjects (3.5).

* Sex: 58.2 percent of controls are male compared to 36.3 percent of depressed subjects (p equals 0.0045).

* Education: 60.9 percent of controls have a college degree, while only 28.8 percent of depressed subjects do (p equals 1.06 times 10 super negative 4).

* Employment: 58.2 percent of controls are employed full-time compared to 33.8 percent of depressed subjects. 18.8 percent of depressed subjects are unemployed compared to 0 percent of controls.

* Childhood Trauma Questionnaire: 43.8 percent of depressed subjects reported trauma compared to 10 percent of controls (p equals 2.1 times 10 super negative 7).

* Medication: In the depressed group, 43.8 percent use antidepressants and 33.8 percent use a combination of antidepressants and other psychotropics; the control group is unmedicated by design.

Navigate back to Table 1..

### Table 2. Long description

The table consists of six columns and four rows. The columns are labeled Model, I C C, R super 2, Pearson’s R, M A E, and R M S E.

* The first data row for the model brainageR shows an I C C of 0.453, R super 2 of 0.206, Pearson’s R of 0.454, M A E of 8.84, and R M S E of 12.4.

* The second data row for the model DeepBrainNet shows an I C C of 0.471, R super 2 of 0.28, Pearson’s R of 0.529, M A E of 9.37, and R M S E of 12.1.

* The third data row for the model pyment shows an I C C of 0.317, R super 2 of 0.107, Pearson’s R of 0.328, M A E of 9.37, and R M S E of 12.0.

A footer note defines the initialisms: I C C stands for intraclass correlation, M A E stands for mean absolute error, and R M S E stands for root mean square error.

Navigate back to Table 2..

### Table 3. Long description

The table is organized into eight sections, each representing a different regression model. The columns are Beta, C I (Confidence Interval), t-value, and P (p-value).

* Group main effect: N = 190, R super 2 = 0.273. Significant predictors include Intercept (Beta 6.36, P < .001), Age (Beta -0.39, P < .001), and Sex (Beta -2.97, P = .014). Group is not significant (P = .646).

* Group subtype main effect: N = 80, R super 2 = 0.142. Significant predictors are Intercept (Beta 4.9, P < .001) and Age (Beta -0.19, P = .012).

* Age * Group interaction effect: N = 190, R super 2 = 0.317. Significant predictors include Intercept (P < .001), Age (Beta -0.54, P < .001), Sex (Beta -3.26, P = .006), and the Age * Group interaction (Beta 0.34, P < .001).

* Age * Group subtype interaction effect: N = 80, R super 2 = 0.150. Only the Intercept is significant (P < .001).

* Childhood trauma main effect: N = 190, R super 2 = 0.275. Significant predictors are Intercept (P < .001), Age (Beta -0.39, P < .001), and Sex (Beta -3.11, P = .008). C T Q is not significant.

* C A R main effect: N = 53, R super 2 = 0.338. Significant predictors are Intercept (P < .001), Age (Beta -0.31, P < .001), and A U C i (Beta -0.01, P = .041).

* Childhood trauma * Group interaction effect: N = 190, R super 2 = 0.286. Significant predictors are Intercept (P < .001), Age (Beta -0.39, P < .001), and Sex (Beta -2.7, P = .026).

* C A R * Group interaction effect: N = 53, R super 2 = 0.369. Significant predictors are Intercept (P = .004), Age (Beta -0.29, P < .001), and A U C i (Beta -0.01, P = .014).

Note: All p-values are corrected using the false discovery rate. Initialisms include A I C (Akaike Information Criterion), B I C (Bayesian Information Criterion), C A R (Cortisol Awakening Response), and C T Q (Childhood Trauma Questionnaire).

Navigate back to Table 3..

### Figure 1. Long description

The x-axis represents Age in years, ranging from 20 to 70. The y-axis represents Brain-P A D in years, ranging from negative 20 to 20. A legend on the right identifies two groups: control in blue and depression in orange.

Both groups show a negative linear correlation where brain-P A D decreases as age increases. The control group, represented by blue dots and a dark blue regression line with a light blue confidence interval, starts with a higher brain-P A D at age 20 and shows a steeper negative slope. The depression group, represented by orange dots and an orange regression line with a light orange confidence interval, starts with a lower brain-P A D at age 20 and exhibits a shallower negative slope. The two regression lines intersect at approximately age 35. Beyond this intersection point, the depression group maintains a higher brain-P A D relative to the control group for the same chronological age.

Navigate back to Figure 1..

### Figure 2. Long description

The x-axis is labeled Cortisol output A U C i with numerical values ranging from negative 250 to 500. The y-axis is labeled Brain dash P A D with numerical values ranging from negative 10 to 30.

A legend on the right identifies two groups: control represented by blue dots and depression represented by orange dots.

The data points are scattered across the center of the plot, mostly between negative 10 and 15 on the y-axis. One outlier blue dot is located at the top left near the y-value of 30.

A solid black regression line shows a linear decrease from left to right, starting at a Brain dash P A D value of approximately 6 at the negative 500 x-intercept and ending near 0 at the 600 x-intercept. A light gray shaded area surrounds the regression line, representing the confidence interval, which widens slightly at both ends of the x-axis.

Navigate back to Figure 2..
