# Supplementary material for: Depression and aging: insights from brain age prediction models
Source: Psychol Med. 2026 Jun 23;56:e204. doi: 10.1017/S0033291726104851 (PMC13319484; doi:10.1017/S0033291726104851)
Supplement: Mitchell et al. supplementary material [file S0033291726104851sup001.docx]

Supplementary Material

**Shared Mechanisms of Depression and Aging: Insights from Brain Age Prediction Models**

Orla Mitchell^*1^, Michael Connaughton^1^, John R Kelly^2^, Andrew Harkin^2^, Darren W Roddy^1^, Monica Aas^3^

1. Department of Psychiatry, Royal College of Surgeons in Ireland, Dublin 2, Ireland
2. Trinity College Institute of Neuroscience, Trinity College Dublin, Dublin 2, Ireland
3. Social, Genetic & Developmental Psychiatry Centre, Institute of Psychiatry, Psychology & Neuroscience, King’s College London, London, United Kingdom

**Corresponding author**

Orla Mitchell

Department of Psychiatry

Royal College of Surgeons in Ireland

Dublin 2, Ireland

K23054625@kcl.ac.uk

[Orlamitchell23@rcsi.ie](mailto:Orlamitchell23@rcsi.ie)

**Table of Contents**

[1. Brain Age Prediction Models 3](#_Toc224120518)

[1.1 Model Description 3](#_Toc224120519)

[BrainageR 3](#_Toc224120520)

[DeepBrainNet 3](#_Toc224120521)

[pyment 3](#_Toc224120522)

[1.2 Pre-processing and Segmentation 3](#_Toc224120523)

[BrainageR 3](#_Toc224120524)

[DeepBrainNet & pyment 4](#_Toc224120525)

[2. Model Selection 4](#_Toc224120526)

[**eFigure 1.** Relationship between chronological age and brain-PAD before and after age-bias correction. 5](#_Toc224120527)

[**eFigure 2.** Distribution of Global Tissue Volumes in brainageR. 7](#_Toc224120528)

[**eFigure 3.** Distribution of Predicted Age Difference by Model 8](#_Toc224120529)

[**eFigure 4.** Predicted age vs. Chronological Age for all three Models 8](#_Toc224120530)

[**eTable 1**. Model performance Assessment Metrics 9](#_Toc224120531)

[**eFigure 5:** Model Validation 9](#_Toc224120532)

[**eFigure 6.** Comparison of DeepBrainNet Skull Stripping Intensity Thresholds 11](#_Toc224120533)

[4. Primary and Secondary Analysis 16](#_Toc224120534)

[**eFigure 7.** Distribution of Cortisol by Group 17](#_Toc224120535)

[**eFigure 8.** Cortisol Brain-PAD correlation 18](#_Toc224120536)

[**eFigure 9.** Distribution of Brain-PAD by Groups 19](#_Toc224120537)

# Brain Age Prediction Models

## 1.1 Model Description

### BrainageR

The brainageR software package (v2.1) (<https://github.com/james-cole/brainageR>) employs Gaussian Process Regression and was developed by Cole et al. (2018). The package spatially normalizes and vectorizes segmented grey matter, white matter, and CSF probability maps generated by SPM12, then applies principal component analysis using the top 435 components as input for brain age prediction. The model was trained on 3,377 healthy individuals (mean age = 40.6 years, SD = 21.4, range = 18-92 years), from seven publicly available datasets. Further details on training and testing datasets can be found in the Biondo et al. (2022) paper.

## DeepBrainNet

DeepBrainNet (https://github.com/vishnubashyam/DeepBrainNet), developed by Bashyam et al. (2020), employs a two-dimensional CNN architecture leveraging ImageNet pre-training. The model divides T1-weighted images into 80 2D slices and predicts brain age based on FreeSurfer-derived features including cortical thickness, surface area, and subcortical volumes. Final predictions represent the median of slice-based age estimates. Training utilized T1-weighted MRI data from 11,729 individuals aged 3-95 (Valdes-Hernandez et al., 2023), with model performance evaluated on an independent test set of 2,739 individuals (Bashyam et al., 2020).

## pyment

The pyment package (<https://github.com/estenhl/pyment-public>) utilizes a Simple Fully Convolutional Network (SFCN) architecture to estimate brain age from 3D T1-weighted MRIs (Peng, Gong, Beckmann, Vedaldi, & Smith, 2021). We selected the SFCN-reg model trained on 53,542 participants aged 3-95 years from 21 non-overlapping publicly available datasets, which employs the base SFCN with a regression prediction head.

## 1.2 Pre-processing and Segmentation

### BrainageR

Following manual inspection for alignment, scans were pre-processed and segmented using SPM12 (https://www.fil.ion.ucl.ac.uk/spm/software/spm12/) according to the brainageR software pipeline. Pre-processing included bias field correction to address intensity inhomogeneities (bias regularisation = 0.001, FWHM = 60 mm, followed by segmentation into grey matter, white matter, and cerebrospinal fluid using SPM12’s segmentation algorithm. To improve inter-subject alignment, segmented tissue maps were registered to a template using DARTEL. The resulting flow fields were used to normalise GM, WM, and CSF maps to MNI space, with images modulated to preserve total tissue volumes and smoothed using a 4mm FWHM Gaussian kernel. Global tissue volumes were computed and exported for quality control assessment through manual inspection and visualisation in RStudio (Figure 1). Outliers were identified using z-score thresholds (|z| > 3) and subjected to additional inspection and sensitivity analyses.

## DeepBrainNet & pyment

For DeepBrainNet and pyment analyses, scans underwent skull-stripping using FSL’s BET tool. Following visual and statistical (Figure 5) comparison of multiple fractional intensity thresholds (0.1, 0.2, 0.3, 0.5), a threshold of 0.2 was determined optimal. Skull-stripped images were linearly registered to the MNI152 1mm template using FSL’s FLIRT with 12 degrees of freedom. Pre-processed scans were visually inspected for spatial alignment, with one participant excluded due to substantial misalignment errors.

Pyment required additional pre-processing steps. Brain extraction generated masks to exclude background voxels, followed by min-max normalisation of voxel intensities to [0, 1] range for consistency with training data. Images were resampled to the required input dimensions (167 × 212 × 160 voxels) using trilinear interpolation, chosen to balance anatomical fidelity whilst avoiding high-frequency artifacts and minimising domain shift relative to model training preprocessing.

# Model Selection

Among the three brain age prediction models, pyment performed the poorest across all the metrics. Whilst brainageR achieved lower mean absolute error (MAE), DeepBrainNet demonstrated superior performance in correlation, R^2^, and ICC values, indicating better capture of age variance and more reliable agreement between predicted and chronological age in the control group (Table 1). Bias analysis revealed that brainageR and pyment were unbiased on average (mean brain-PAD not significantly different form zero), whereas DeepBrainNet systematically overestimated age by approximately 5.5 years (95% CI: 3.41–7.49, p < .001; Figure 2). Despite this systematic bias, we selected DeepBrainNet as the optimal model because subsequent analyses include mean centred age as a covariate, meaning this systematic offset does not compromise group comparisons. The correlations between predicted and chronological age as well as model validation for all three models are shown in Figure 3.

To evaluate whether the regression to the mean bias influenced our findings, we conducted a sensitivity analysis applying the age-bias correction procedure described by Behesti et al., (2019), which removes systematic age related residuals. As expected, this eliminated the association between chronological age and brain-PAD in controls and importantly, when the primary regression models were repeated using these corrected values, the Age x Diagnosis interaction remained unchanged (β = 0.34, p = .0007) (eFigure 1). This suggests that the observed group difference in the age-brain-PAD association is unlikely to be solely explained by the regression to the mean phenomenon.

## **eFigure 1.** Relationship between chronological age and brain-PAD before and after age-bias correction.

*
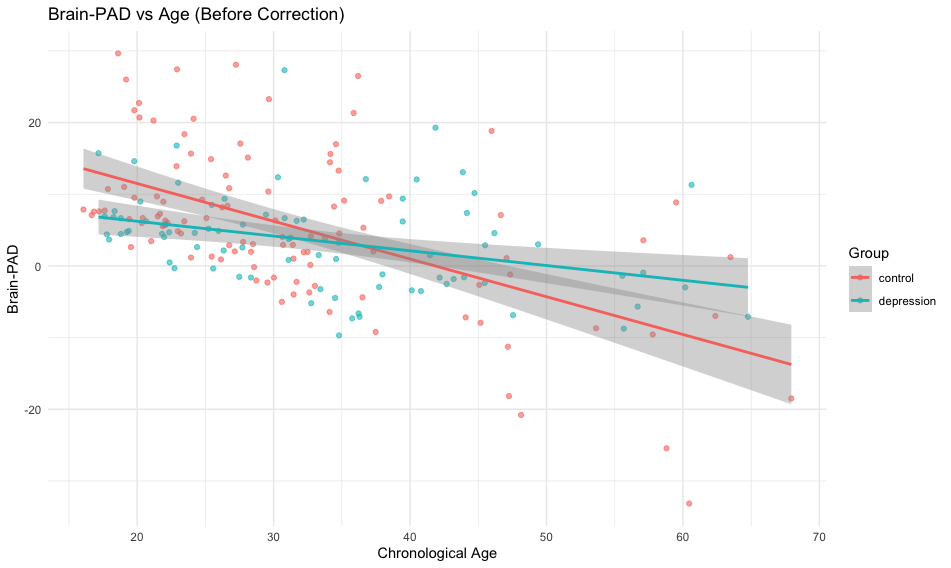
*(A)

*
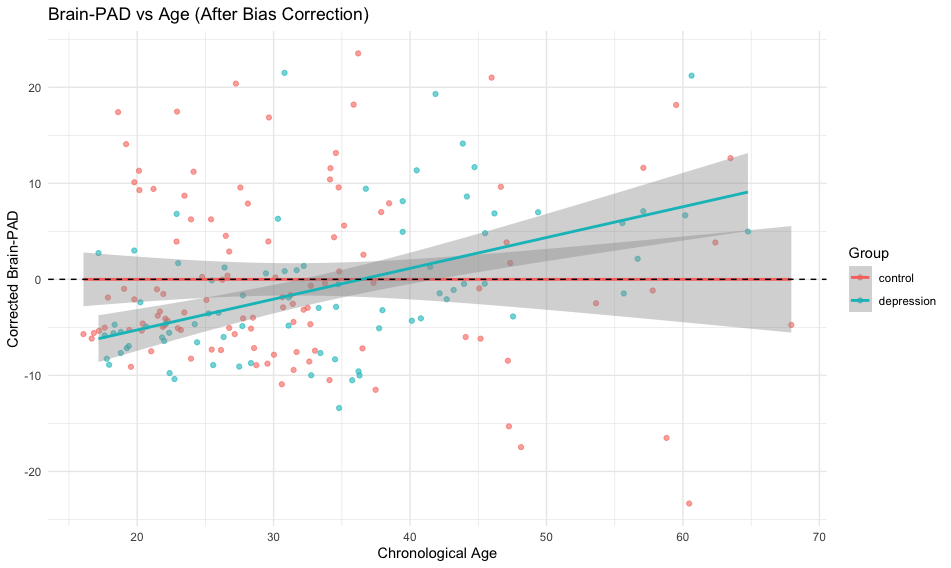
*(B)

***Legend:*** Scatterplots showing the relationship between chronological age and brain-predicted age difference (brain-PAD) before (Panel A) and after (Panel B) applying the age-bias correction procedure described by Beheshti et al. (2019). Prior to correction, brain-PAD exhibited a significant negative association with chronological age, consistent with the regression to the mean pattern. Following correction, this association was removed in the control group, indicating successful elimination of systematic age-dependent bias.

1. Sensitivity Analyses

Sensitivity analyses using continuous CTQ total scores, continuous subtype scores, and abuse and neglect related composites yielded comparable results. No continuous childhood adversity measure was significantly associated with brain-PAD after correction for multiple comparisons (all FDR-corrected p > .05). Neither abuse related nor neglect related composite scores was significantly associated with brain-PAD. Among individual subtype analyses, physical neglect showed a nominal association with brain-PAD (β = 2.46, p = 0.026), but this did not survive correction for multiple comparisons (FDR-corrected p = 0.058). We found no correlation between CTQ measures and baseline cortisol (p = 0.14) or CAR (p = 0.54). Additional sensitivity analyses adjusting for education, employment status, and marital status did not materially alter the results.

##

## **
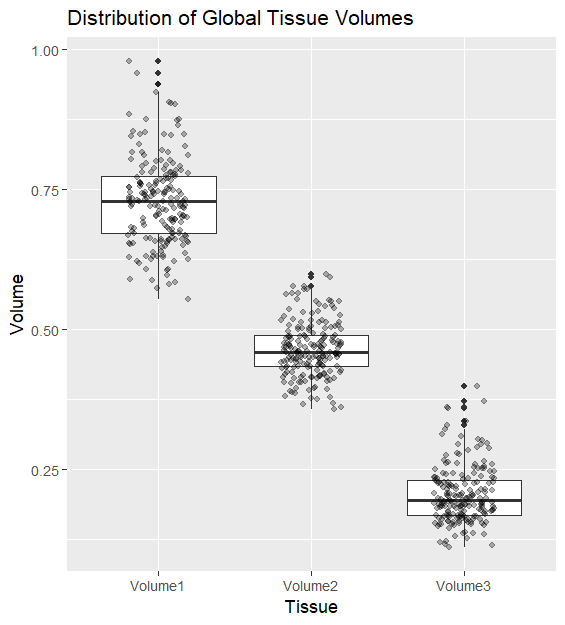
eFigure 2.** Distribution of Global Tissue Volumes in brainageR.

***Legend:*** Volume 1; Grey Matter, Volume 2; White Matter, Volume 3; Cerebrospinal Fluid.

## **
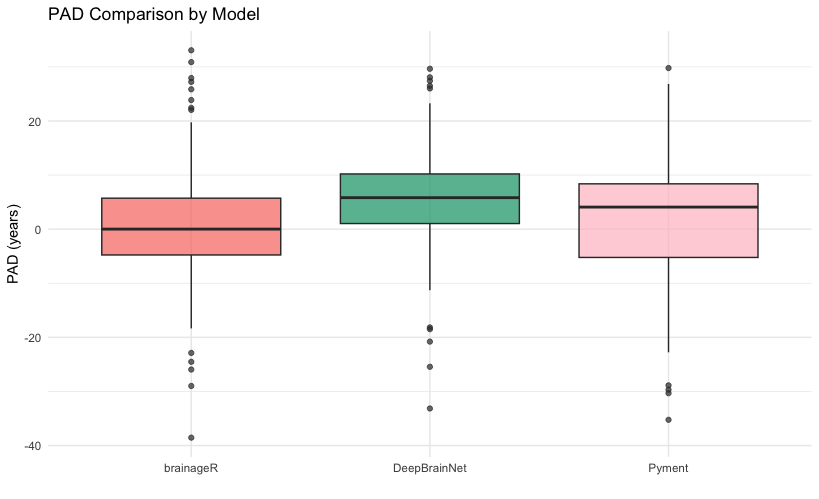
eFigure 3.** Distribution of Predicted Age Difference by Model

## **
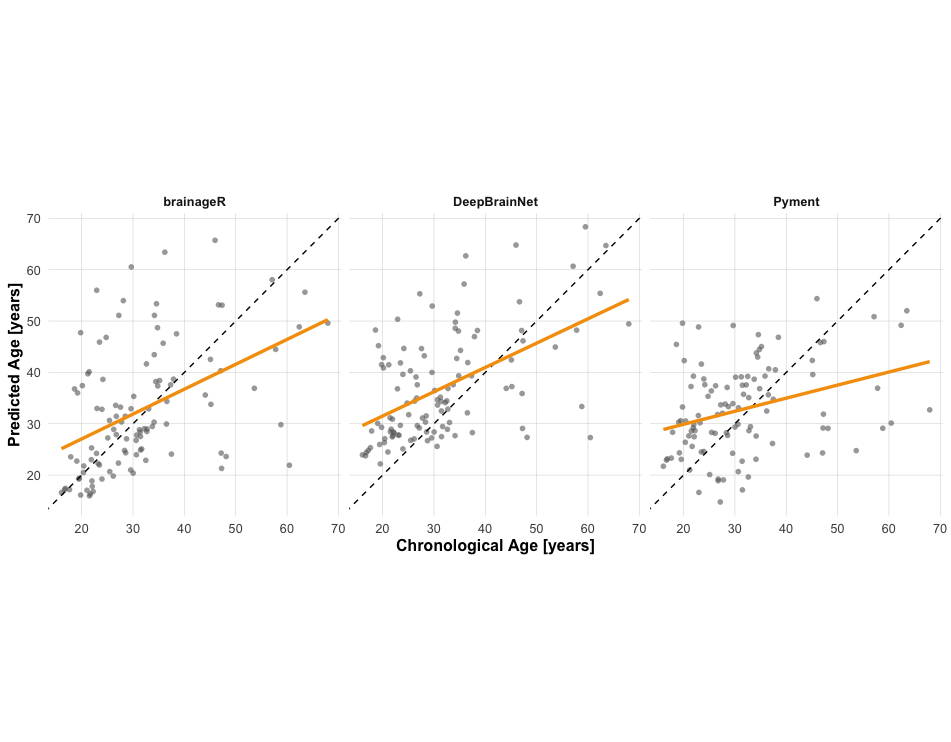
eFigure 4.** Predicted age vs. Chronological Age for all three Models

## **eTable 1**. Model performance Assessment Metrics

| Model | ICC | R^2^ | Pearsons R | MAE | RMSE |
| --- | --- | --- | --- | --- | --- |
|  |  |  |  |  |  |
| brainageR | 0.453 | 0.206 | 0.454 | 8.84 | 12.4 |
| DeepBrainNet | 0.471 | 0.28 | 0.529 | 9.37 | 12.1 |
| pyment | 0.317 | 0.107 | 0.328 | 9.37 | 12.0 |

***Legend***: ICC; intraclass correlation, MAE; mean absolute error, RMSE; root mean square error.

## **eFigure 5:** Model Validation


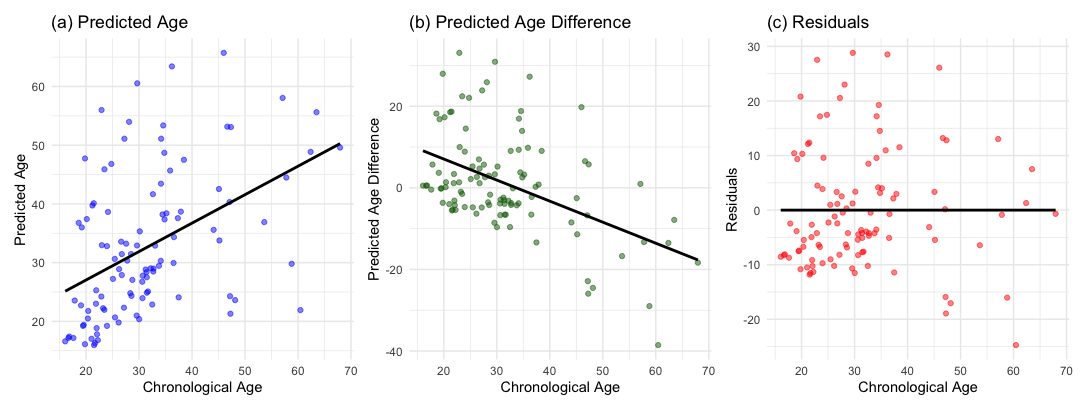
(A)


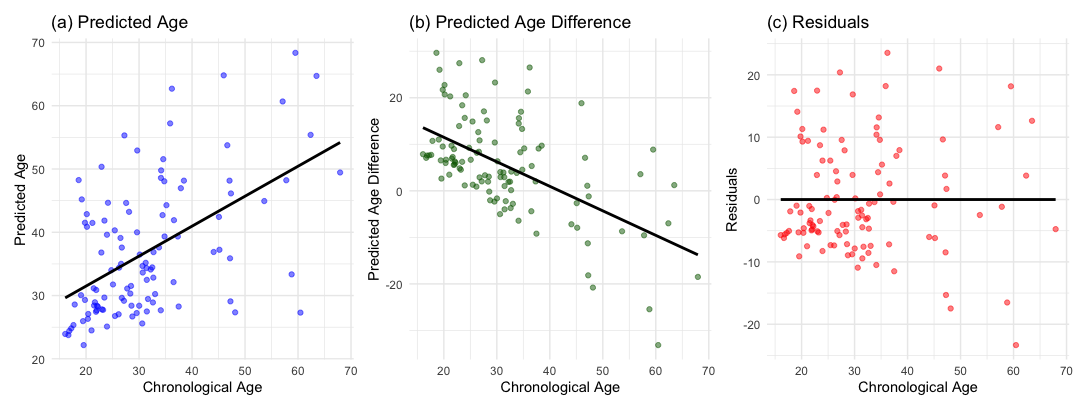
(B)


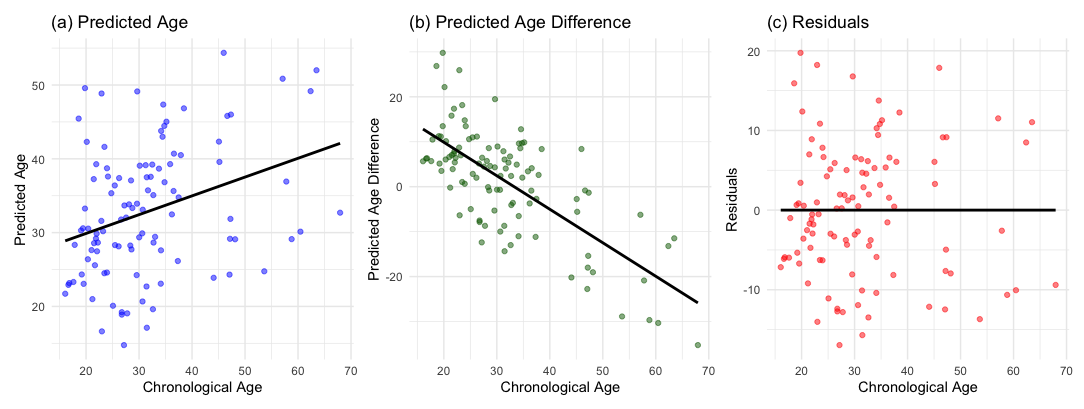
(C)

***Legend*:**  Model validation in control subjects. DeepBrainNet is the most accurate in absolute prediction and has the least regression to the mean bias; brainageR performs well but shows stronger regression to the mean bias. (A) brainageR: (a) Predicted vs chronological age (R = 0.69, R² = 0.48, p < .001), (b) PAD vs chronological age (R = −0.69, R² = 0.47, p < .001), (c) residuals of PAD ~ age vs age; (B) DeepBrainNet: (a) Predicted vs chronological age (R = 0.76, R² = 0.58, p < .001), (b) PAD vs chronological age (R = −0.51, R² = 0.26, p = .009), (c) residuals of PAD ~ age vs age; (C) Pyment: (a) Predicted vs chronological age (R = 0.13, R² = 0.02, p = .54), (b) PAD vs chronological age (R = −0.73, R² = 0.53, p < .001), (c) residuals of PAD ~ age vs age.

## **eFigure 6.** Comparison of DeepBrainNet Skull Stripping Intensity Thresholds

A


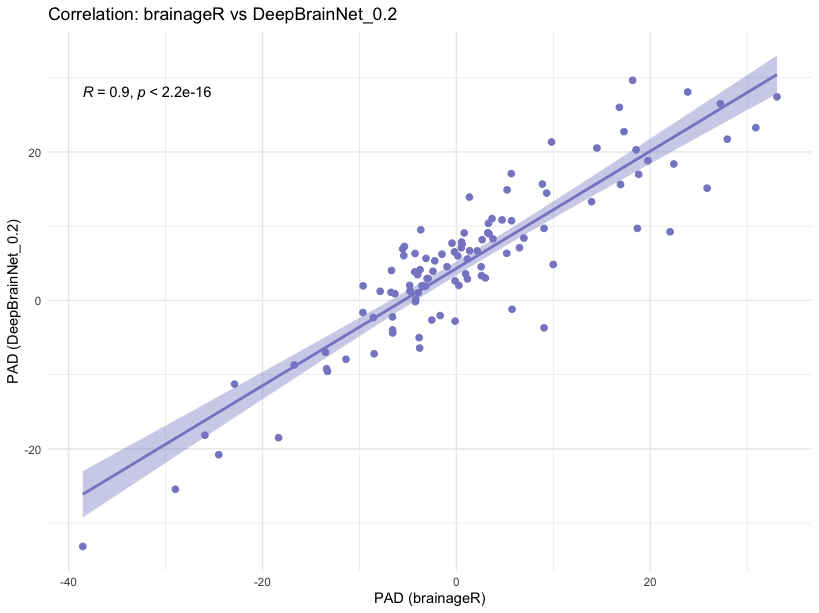

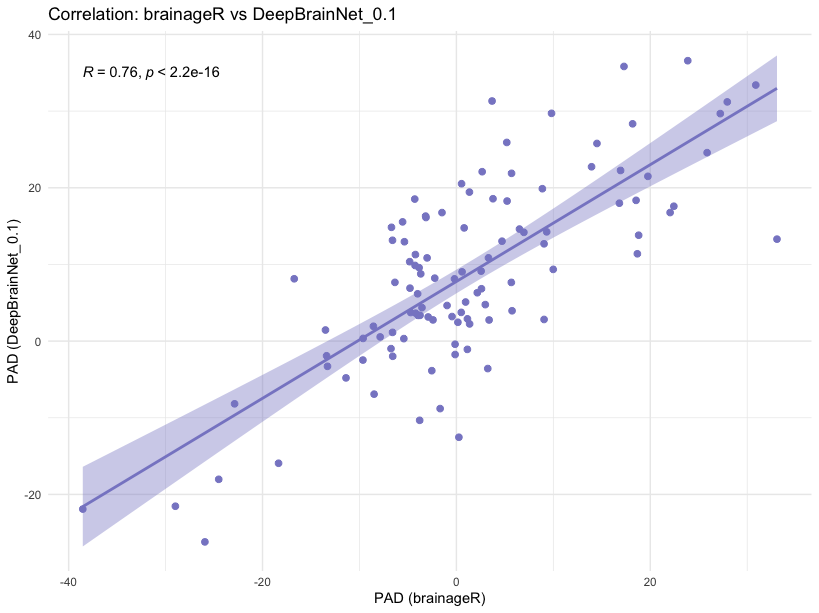


B


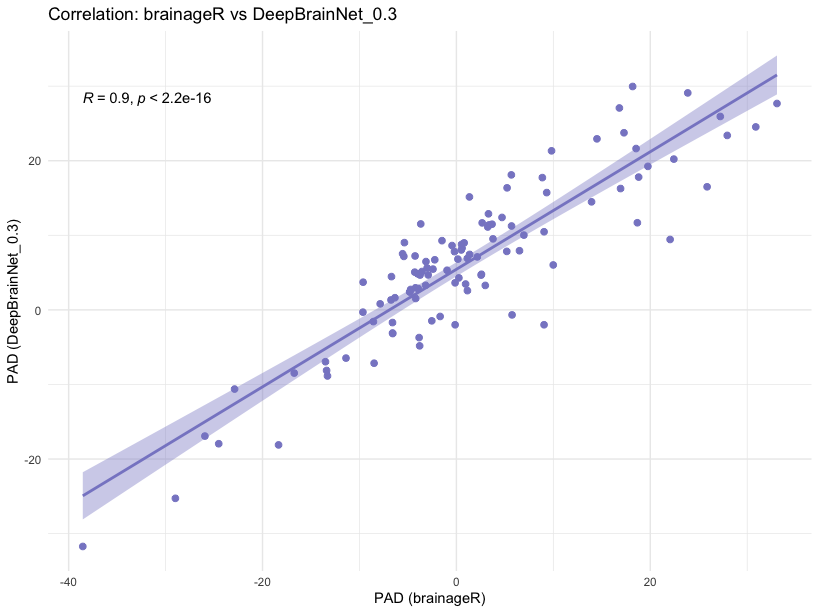

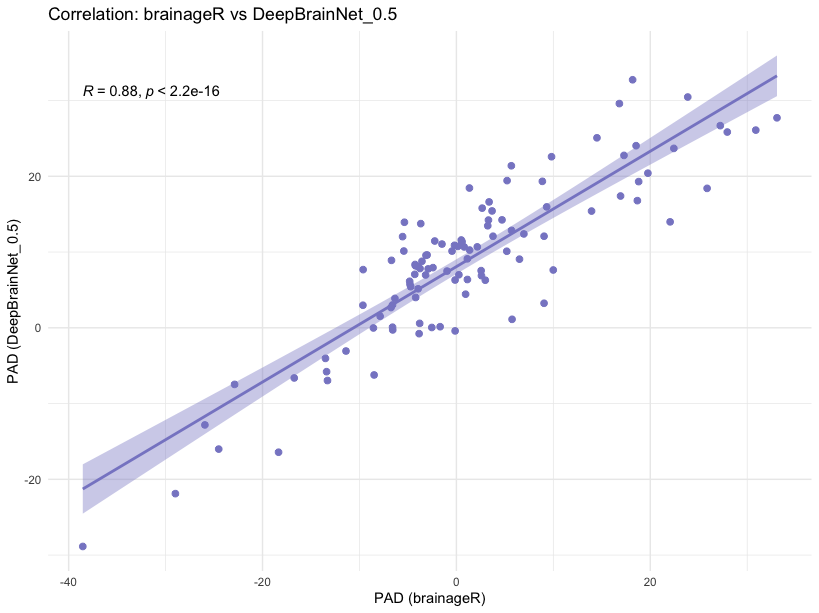


D

C


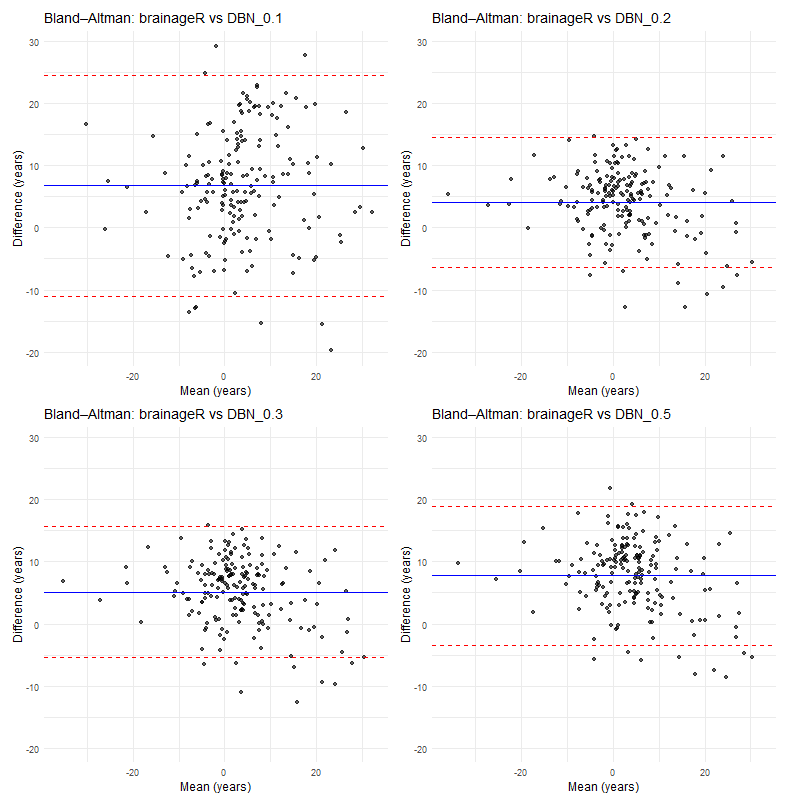


E

F


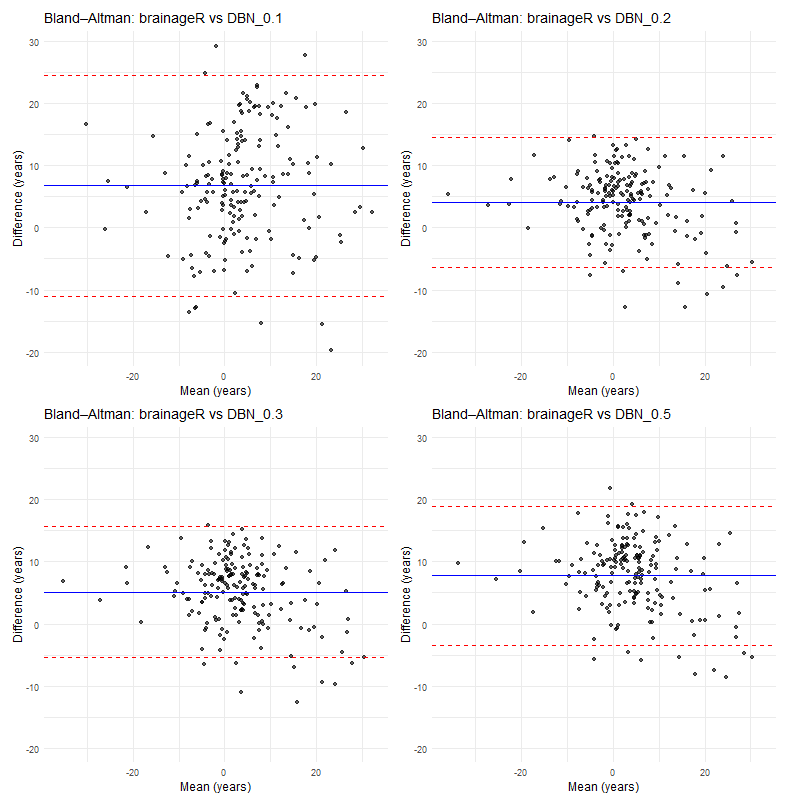


G

H

***Legend***: Based on visual and statistical inspection, a fractional intensity threshold of 0.2 was selected for skull stripping for DeepBrainNet and pyment pre-processing. (A) Correlation of brainageR brain-PAD and DeepBrainNet brain-PAD with skull stripping with fractional intensity of 0.1, (B) Correlation of brainageR brain-PAD and DeepBrainNet brain-PAD with skull stripping with fractional intensity of 0.2, (C) Correlation of brainageR brain-PAD and DeepBrainNet brain-PAD with skull stripping with fractional intensity of 0.3, (D) Correlation of brainageR brain-PAD and DeepBrainNet brain-PAD with skull stripping with fractional intensity of 0.5, (E) Bland-Altman plot showing the agreement between brainageR brain-PAD and DeepBrainNet brain-PAD with skull stripping with fractional intensity 0.1, (F) Bland-Altman plot showing the agreement between brainageR brain-PAD and DeepBrainNet brain-PAD with skull stripping with fractional intensity 0.2, (G) Bland-Altman plot showing the agreement between brainageR brain-PAD and DeepBrainNet brain-PAD with skull stripping with fractional intensity 0.3, (H) Bland-Altman plot showing the agreement between brainageR brain-PAD and DeepBrainNet brain-PAD with skull stripping with fractional intensity 0.5. **DBN_0.1**; DeepBrainNet Skull Stipped with Fractional Intensity Threshold 0.1, **DBN_0.2**; DeepBrainNet Skull Stripped with Fractional Intensity Threshold 0.2, **DBN_0.3**; DeepBrainNet Skull Stripped with Fractional Intensity Threshold 0.3, **DBN_0.5**; DeepBrainNet Skull Stripped with Fractional Intensity Threshold 0.5.

# Primary and Secondary Analysis

Our primary analysis examined brain-predicted age difference (brain-PAD = predicted age - chronological age) between depression and control groups. Following literature recommendations (Le et al., 2018; Liang, Zhang, & Niu, 2019), age was included as a covariate in all models, alongside sex given its established influence on brain aging. Secondly, we stratified the depression group by episode type (first episode vs. recurrent) to test whether illness chronicity influences brain aging and fitted interaction models to examine whether age-brain-PAD relationships differed by diagnosis. Ethnicity was tested as a covariate (Chee, Zheng, Goh, Park, & Sutton, 2011; Isamah et al., 2010; Kang et al., 2020) but did not significantly improve model fit and was excluded for parsimony. Each model is outlined below.

Primary Analysis:

**Diagnosis Main Effect: Brain-PAD ∼ age + sex + diagnosis**

**Diagnosis Subtype Main Effect: Brain-PAD ∼ age + sex + diagnosis subtype**

**Age x Diagnosis Interaction Effect: Brain-PAD ∼ age*diagnosis + sex**

**Age x Diagnosis Subtype Interaction Effect: Brain-PAD ∼ age*diagnosis subtype + sex**

Secondary Analysis:

**Childhood Trauma Main Effect: Brain-PAD ∼ age + sex + CTQ**

**CAR Main Effect: Brain-PAD ∼ age + sex + AUCi**

**Childhood Trauma x Diagnosis Interaction Effect: Brain-PAD ∼ CTQ*diagnosis + age + sex**

**CAR x Diagnosis Interaction Effect: Brain-PAD ∼ AUCi*diagnosis + age + sex**

Evidence in the literature suggest that taking antidepressant or other psychotropic medication can have differential effects on brain structure (Frodl et al., 2008). Within our sample, all controls were unmedicated, whilst participants with depression were on various medication types. Therefore, including medication status in the full models would introduce the issue of collinearity, potentially leading the model to drop terms or produce unstable estimates. To address this, we conducted a sensitivity analysis within the depression group to examine whether medication status was associated with differences in cortisol output (AUCi) or brain-PAD. For cortisol (AUCi), both ANOVA (F(3,50) = 0.318, p = 0.813) and Kruskal-Wallis tests (χ² = 1.51, df = 3, p = 0.680) indicated no significant differences between medication groups. For brain-PAD, ANOVA showed no significant differences (F(3,182) = 2.06, p = 0.107).

## **
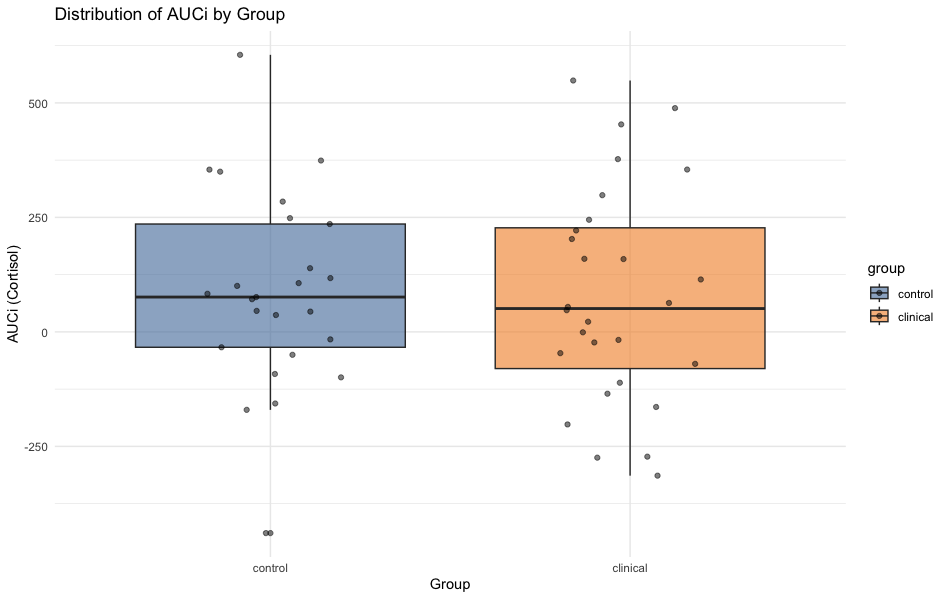
eFigure 7.** Distribution of Cortisol by Group

***Legend*: AUCi**; Area Under Curve with respect to increase

## **
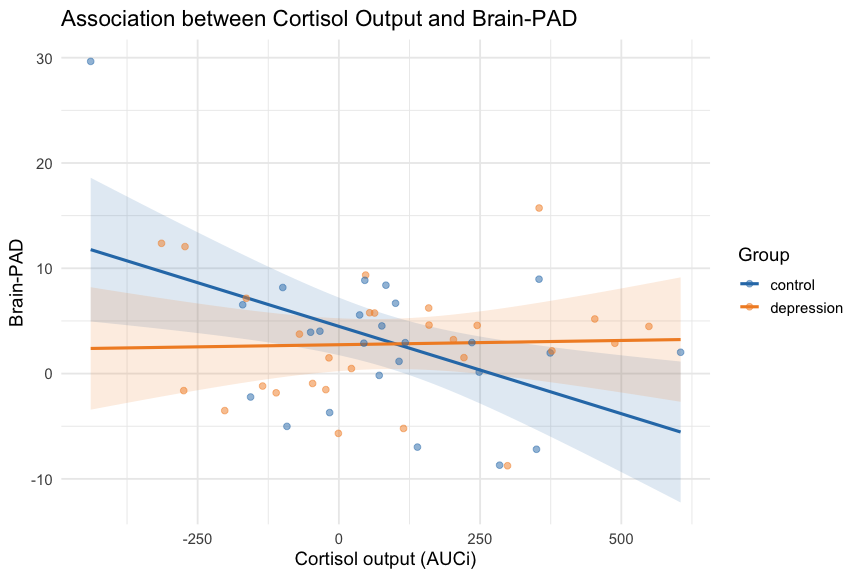
eFigure 8.** Cortisol Brain-PAD correlation

***Legend*: AUCi**; Area Under Curve with respect to increase

## **eFigure 9.** Distribution of Brain-PAD by Groups

***
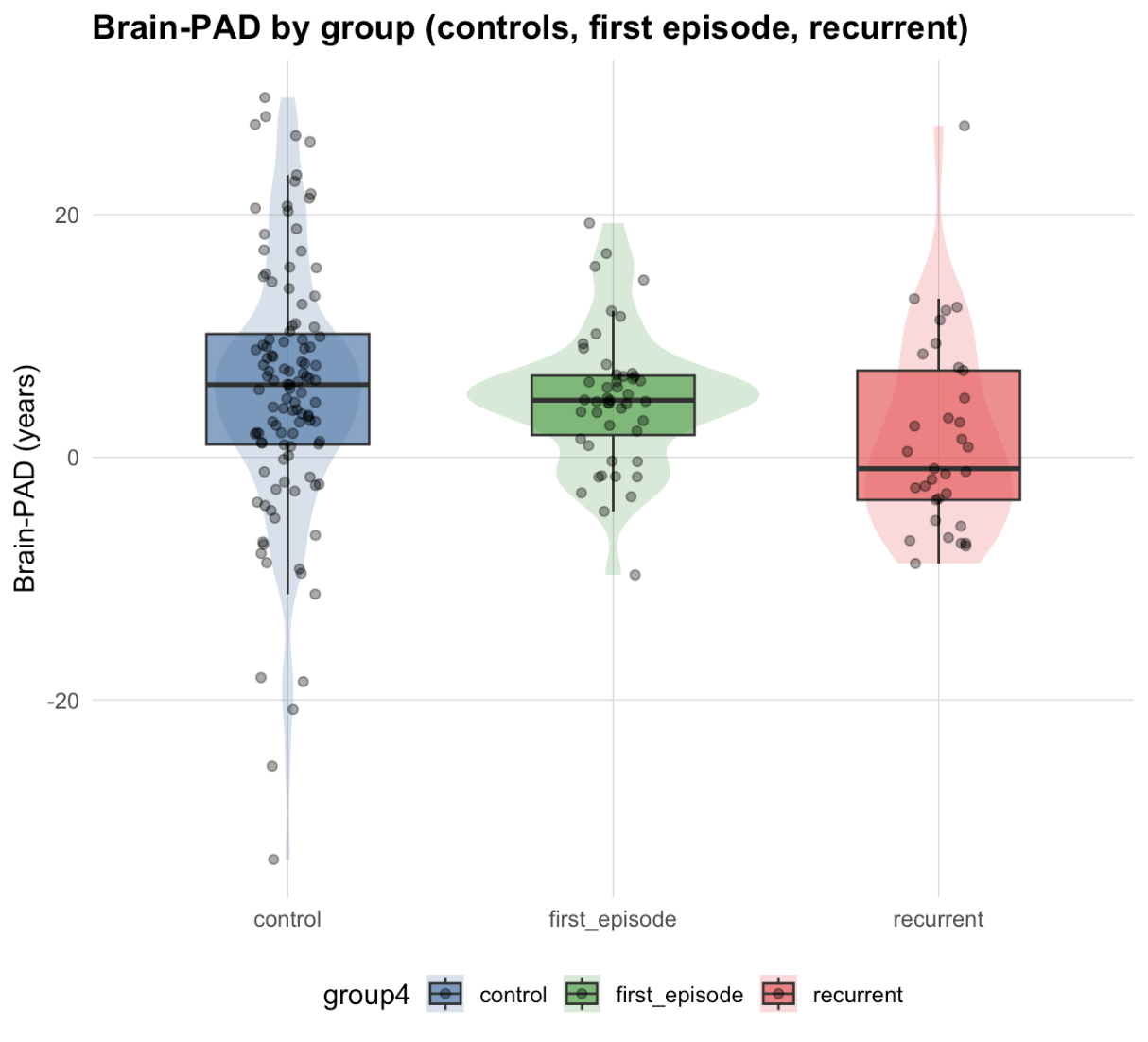
***

Bashyam, V. M., Erus, G., Doshi, J., Habes, M., Nasrallah, I., Truelove-Hill, M., . . . Davatzikos, C. (2020). MRI signatures of brain age and disease over the lifespan based on a deep brain network and 14 468 individuals worldwide. *Brain, 143*(7), 2312-2324. doi:10.1093/brain/awaa160

Beheshti, I., Nugent, S., Potvin, O., & Duchesne, S. (2019). Bias-adjustment in neuroimaging-based brain age frameworks: A robust scheme. *NeuroImage: Clinical, 24*, 102063. doi:<https://doi.org/10.1016/j.nicl.2019.102063>

Biondo, F., Jewell, A., Pritchard, M., Aarsland, D., Steves, C. J., Mueller, C., & Cole, J. H. (2022). Brain-age is associated with progression to dementia in memory clinic patients. *NeuroImage: Clinical, 36*, 103175. doi:<https://doi.org/10.1016/j.nicl.2022.103175>

Chee, M. W., Zheng, H., Goh, J. O., Park, D., & Sutton, B. P. (2011). Brain structure in young and old East Asians and Westerners: comparisons of structural volume and cortical thickness. *J Cogn Neurosci, 23*(5), 1065-1079. doi:10.1162/jocn.2010.21513

Cole, J. H., Ritchie, S. J., Bastin, M. E., Valdés Hernández, M. C., Muñoz Maniega, S., Royle, N., . . . Deary, I. J. (2018). Brain age predicts mortality. *Mol Psychiatry, 23*(5), 1385-1392. doi:10.1038/mp.2017.62

Frodl, T. S., Koutsouleris, N., Bottlender, R., Born, C., Jäger, M., Scupin, I., . . . Meisenzahl, E. M. (2008). Depression-related variation in brain morphology over 3 years: effects of stress? *Arch Gen Psychiatry, 65*(10), 1156-1165. doi:10.1001/archpsyc.65.10.1156

Isamah, N., Faison, W., Payne, M. E., MacFall, J., Steffens, D. C., Beyer, J. L., . . . Taylor, W. D. (2010). Variability in frontotemporal brain structure: the importance of recruitment of African Americans in neuroscience research. *PLoS One, 5*(10), e13642. doi:10.1371/journal.pone.0013642

Kang, D. W., Wang, S. M., Na, H. R., Park, S. Y., Kim, N. Y., Lee, C. U., . . . Lim, H. K. (2020). Differences in cortical structure between cognitively normal East Asian and Caucasian older adults: a surface-based morphometry study. *Sci Rep, 10*(1), 20905. doi:10.1038/s41598-020-77848-8

Le, T. T., Kuplicki, R. T., McKinney, B. A., Yeh, H. W., Thompson, W. K., & Paulus, M. P. (2018). A Nonlinear Simulation Framework Supports Adjusting for Age When Analyzing BrainAGE. *Front Aging Neurosci, 10*, 317. doi:10.3389/fnagi.2018.00317

Liang, H., Zhang, F., & Niu, X. (2019). Investigating systematic bias in brain age estimation with application to post-traumatic stress disorders. *Hum Brain Mapp, 40*(11), 3143-3152. doi:10.1002/hbm.24588

Peng, H., Gong, W., Beckmann, C. F., Vedaldi, A., & Smith, S. M. (2021). Accurate brain age prediction with lightweight deep neural networks. *Med Image Anal, 68*, 101871. doi:10.1016/j.media.2020.101871

Valdes-Hernandez, P. A., Laffitte Nodarse, C., Johnson, A. J., Montesino-Goicolea, S., Bashyam, V., Davatzikos, C., . . . Cruz-Almeida, Y. (2023). Brain-predicted age difference estimated using DeepBrainNet is significantly associated with pain and function-a multi-institutional and multiscanner study. *Pain, 164*(12), 2822-2838. doi:10.1097/j.pain.0000000000002984
